# Coronary artery calcification, cardiovascular events, and death: a prospective cohort study of incident patients on hemodialysis

**DOI:** 10.1186/s40697-015-0065-6

**Published:** 2015-08-12

**Authors:** Trevor J. Wilkieson, M. Omair Rahman, Azim S. Gangji, Maurice Voss, Alistair J. Ingram, Nischal Ranganath, Charlie H. Goldsmith, Cathy Z. Kotsamanes, Mark A. Crowther, Christian G. Rabbat, Catherine M. Clase

**Affiliations:** Department of Medicine, McMaster University, 50 Charlton Avenue East, Hamilton, L8N4A6 ON Canada; Department of Clinical Epidemiology and Biostatistics, McMaster University, Hamilton, Canada; Department of Radiology, McMaster University, Hamilton, Canada; Faculty of Health Sciences, Simon Fraser University and Arthritis Research Centre of Canada, Richmond, Canada

**Keywords:** End stage renal disease, Coronary artery calcification, Hemodialysis, Death, Myocardial infarction, Cardiovascular outcome

## Abstract

**Background:**

Coronary calcification in patients with end-stage renal disease (ESRD) is associated with an increased risk of cardiovascular outcomes and death from all causes. Previous evidence has been limited by short follow-up periods and inclusion of a heterogeneous cluster of events in the primary analyses.

**Objective:**

To describe coronary calcification in patients incident to ESRD, and to identify whether calcification predicts vascular events or death.

**Design:**

Prospective substudy of an inception cohort.

**Setting:**

Tertiary care haemodialysis centre in Ontario (St Joseph’s Healthcare Hamilton).

**Participants:**

Patients starting haemodialysis who were new to ESRD.

**Measurements:**

At baseline, clinical characterization and spiral computed tomography (CT) to score coronary calcification by the Agatston-Janowitz 130 scoring method. A primary outcome composite of adjudicated stroke, myocardial infarction, or death.

**Methods:**

We followed patients prospectively to identify the relationship between cardiac calcification and subsequent stroke, myocardial infarction, or death, using Cox regression.

**Results:**

We recruited 248 patients in 3 centres to our main study, which required only biochemical markers. Of these 164 were at St Joseph’s healthcare, and eligible to participate in the substudy; of these, 51 completed CT scanning (31 %). Median follow up was 26 months (Q_1_, Q_3_: 14, 34). The primary outcome occurred in 16 patients; 11 in the group above the median and 5 in the group below (*p* = 0.086). There were 26 primary outcomes in 16 patients; 20 (77 %) events in the group above the coronary calcification median and 6 (23 %) in the group below (*p* = 0.006). There were 10 deaths; 8 in the group above the median compared with 2 in the group below (*p* = 0.04). The hazard ratios for coronary calcification above, compared with below the median, for the primary outcome composite were 2.5 (95 % CI 0.87, 7.3; *p* = 0.09) and 1.7 (95 % CI 0.55, 5.4; *p* = 0.4), unadjusted and adjusted for age, respectively. For death, the hazard ratios were 4.6 (95 % CI 0.98, 21.96; *p* = 0.054) and 2.4 (95 % CI 0.45, 12.97; *p* = 0.3) respectively.

**Limitations:**

We were limited by a small sample size and a small number of events.

**Conclusions:**

Respondent burden is high for additional testing around the initiation of dialysis. High coronary calcification in patients new to ESRD has a tendency to predict cardiovascular outcomes and death, though effects are attenuated when adjusted for age.

## Abrégé

**Contexte:**

La calcification de l'artère coronaire chez les patients atteints d’insuffisance rénale terminale (IRT) est associée à un risque accru de troubles cardiovasculaires et de mortalité, toutes causes confondues. Les données précédemment recueillies se limitaient à un suivi de courte durée, de même qu’à l’inclusion de séries d’accidents non liés lors de l’analyse préliminaire.

**Objectifs:**

Décrire la calcification de l'artère coronaire chez les patients atteints d’IRT et déterminer si la calcification de l'artère coronaire peut prédire des accidents vasculaires et la mort.

**Type d’étude:**

Sous-étude prospective de cohorte selon le mode d'installation.

**Cadre:**

Une unité de soins tertiaires en dialyse, en Ontario (St Joseph’s Healthcare Hamilton).

**Participants:**

Des patients qui sont nouvellement atteints d’IRT et qui entament une hémodialyse.

**Mesures:**

En début de traitement, une caractérisation clinique et une tomodensitométrie (TDM) hélicoïdale qui permettent de mesurer la calcification de l'artère coronaire sur 130, selon l’échelle d’Agatston-Janowitz. L’indicateur principal des résultats comprend l’AVC, l’infarctus du myocarde ou la mort.

**Méthodes:**

Nous avons suivi les patients de manière prospective, afin de cibler la relation entre la calcification de l'artère coronaire et l’AVC, l’infarctus du myocarde ou la mort subséquente, en utilisant la régression de Cox.

**Résultats:**

Nous avons recruté 248 patients dans trois unités, dans le cadre de l’étude principale, qui ne requérait que des biomarqueurs chimiques. De ces patients, 164 étaient de St Joseph’s Healthcare, et étaient admissibles à la sous-étude; 51 avaient effectué une tomographie par ordinateur (31 %). Le suivi médian s’étendait sur 26 mois (Q1, Q3: 14, 34). L’indicateur principal a été observé chez 16 patients; 11 dans le groupe se trouvant au-dessus de la médiane, et 5 dans le groupe inférieur (*p*?=?0,086). On a observé 26 indicateurs principaux chez 16 patients; 20 (77 %) accidents dans le groupe se trouvant au-dessus de la médiane en ce qui a trait à la calcification et 6 (23 %) dans le groupe inférieur (*p*?=?0,006). Il y a eu 10 décès; 8 dans le groupe se trouvant au-dessus de la médiane et 2 dans le groupe inférieur (*p*?=?0,04). Les taux de risque de calcification de l'artère coronaire se trouvant au-dessus et sous la médiane, pour les indicateurs principaux, étaient respectivement de 2,5 (95 % IC 0,98; 21,96; *p*?=?0,054) et 2,4 (95 % IC 0,45, 12,97; *p*?=?0,3).

**Limites de l’étude:**

Nous avons été limités par la taille restreinte de l’échantillon, de même que par le petit nombre d’accidents.

**Conclusion:**

Le fardeau du répondant repose sur des examens supplémentaires au moment de commencer la dialyse. Un fort taux de calcification de l’artère coronaire chez les patients nouvellement atteints d’IRT tend à prédire des accidents cardiovasculaires et la mort, bien que les effets soient atténués après révision en fonction de l'âge.

## Background

End-stage renal disease (ESRD) affects over 36,000 Canadians, over 546,000 patients in the US, and is currently an international health problem; the majority are treated with hemodialysis (HD) [1, 2]. Cardiovascular death is the most common cause of death in patients with ESRD [3]. Low glomerular filtration rate causes impaired phosphate excretion which may lead to excess secretion of fibroblast growth factor 23 (FGF23). FGF23 may cause reduced 1,25(OH)_2_D, which in turn is implicated in hyperplasia of the parathyroid gland and increased parathyroid hormone (PTH) [4]. These and other factors lead to an active process of calcification of atherosclerotic plaque and of the media of large vessels, including the coronary arteries [5]. Cross sectional evidence has shown that in patients with kidney disease, high coronary calcification scores correlate with many risk factors for death including older age, diabetes, HD vintage, and lower estimated glomerular filtration rate [6–8]. Baseline serum alkaline phosphatase, PTH, and serum calcium also tend to be associated with higher coronary calcification scores in patients on HD [7–9].

Prospective studies have shown that patients on maintenance HD with high coronary calcification have an increased risk of cardiovascular events and death from all causes compared with those with low or no coronary calcification [8–11]. Similar findings were seen in patients with chronic kidney disease (CKD) who were not on HD [12]. Evidence is limited by short follow-up periods [8–10] as well as the inclusion of events of varying severity and clinical importance in a single composite outcome [8, 12]. Some studies have not explicitly adjudicated subjective outcomes [8, 10, 12], or included all patients enrolled in the primary analysis [9, 10].

Two previous prospective studies enrolled incident patients on hemodialysis [13–15], but did not report cardiovascular outcomes. We aimed to conduct a prospective evaluation of an incident cohort of patients starting hemodialysis as their first ESRD treatment modality, and to examine the patients’ coronary calcification scores as a predictor for a rigorously defined and adjudicated homogenous cluster of outcomes: stroke, myocardial infarction, and death from all causes.

## Methods

This was a sub-study of the Canadian Longitudinal Thrombosis in End-Stage Renal Disease (LONG) study, which was a multi-centre prospective cohort involving 4 HD centres in Ontario. We studied those patients recruited from the St Joseph’s Healthcare Hamilton (SJHH) outpatient dialysis centre who consented to a spiral computed tomography (CT) scan. (Spiral CT was not available in the other centres at the time.) We recruited patients with ESRD between October 2004 and October 2007, and followed them until May 2008. (Funding problems, and interruptions in the continuity of investigators and staff prevented our following the patients longer, and delayed our analysis and publication of the data.) Inclusion criteria were the following: informed consent, at least 18 years of age, and on incident HD, which was defined as being on HD between 30 and 90 days. Exclusion criteria were: anticipated renal recovery, in the intensive care unit throughout the recruitment period, scheduled live-donor kidney transplantation within 6 months, difficulty in communication due to language, cognitive disability, speech or hearing impairments, or lack of informed consent. For this calcification substudy there were further exclusions of prior stenting and valve replacement, and current heart rate above 100. This study was approved by the St Joseph’s Healthcare Hamilton research ethics board.

The clinical assessment consisted of patient interviews, physical examinations, and review of in-patient and out-patient records. We characterized patients in terms of age, sex, diabetes, blood pressure (mean of three readings sitting, pre-midweek dialysis), and baseline cardiovascular events including myocardial infarction, coronary artery bypass graft, percutaneous coronary intervention, atrial fibrillation, deep vein thrombosis, and pulmonary embolism. We documented medications (including type of phosphate binder, warfarin use, and active vitamin D compounds) along with laboratory investigations (serum calcium, phosphorus, albumin, alkaline phosphatase, glucose, haemoglobin A1c (HbA1C), haemoglobin, C-reactive protein, uric acid, intact PTH, urinary reduction ratio, leukocytes, and platelets). Laboratory testing was performed before a mid-week dialysis session.

The primary outcome for the present study was a composite of stroke, myocardial infarction, or death. The secondary outcomes included event rate and cause of death (cardiovascular or from all other causes). Two physicians adjudicated each event according to prespecified definitions and reached consensus on disagreements.

### Calcification scoring

We determined coronary calcification scores using a spiral CT scan. Spiral CT has been shown to have a low intra-reader variability of 0.9 % (0 % median) in examining patients with ESRD for coronary calcification [16]. Coronary calcification was scored according to the Agatston-Janowitz 130 scoring method, in which the area of each calcified lesion that has peak attenuation greater than 130 Hounsfield units (HU) is multiplied by a density factor [17]. If between 130 and 199 HU, 200 and 299 HU, 300 and 399 HU, or > 400 HU, the density factor is 1, 2, 3, or 4, respectively [17]. Readings were performed by one experienced radiologist (MV), blinded to baseline clinical data. Researchers and clinicians were blinded to the results for the duration of the study so that the calcification score could not influence care.

### Statistical analysis

We made an a priori decision to analyze patients grouped as above, or below, the coronary calcification score median. We calculated the mean and standard deviation (SD) for all baseline continuous variables and the frequency and percentage of all categorical variables. We compared continuous variables at baseline across groups with t tests and compared categorical variables using chi squared. In our primary analysis we used adjusted and unadjusted survival analyses to measure the association between a coronary calcification score above or below the median and the occurrence of a primary outcome, censoring at the end of the study or if patients moved away or received renal transplants. (We did not censor at transfer to peritoneal dialysis). In our secondary analyses we measured the associations between coronary calcification group and each component of the event cluster. We used the Kaplan-Meier method to create and graph survival curves, and Cox regression, both unadjusted and adjusted for age to assess differences between groups. We compared rates of events per person-time using Poisson-based methods. All analyses were conducted using SPSS version 18.0 for Windows (Chicago, IL, USA).

## Results

Of the 246 patients recruited for the LONG study, 164 were from SJHH and 51 (31 %) of these patients consented to a spiral CT. Median follow up time was 26 months (Q_1_, Q_3_: 14, 34). There was no loss to follow up.

Mean patient age was 59 years (SD 13.4) old, 29 % were male; the most common cause of ESRD was diabetes (61 %). The median coronary calcification score in the sample was 642. Patients with coronary calcification scores above the median (n = 26) were older, had higher serum albumin levels, and had had more deep vein thrombosis and pulmonary embolism events compared with those below the median (n = 25) (Table 1). There were no significant differences between groups in serum calcium, serum phosphorus, alkaline phosphatase, C-reactive protein, international normalized ratio, urea reduction ratio, presence of liver disease, presence of diabetes, blood pressure, or warfarin use.

**Table 1:** Baseline characteristics of participants

Baseline Characteristics	Coronary calcification below median (n = 25)	Coronary calcification above median (n = 26)	Total (N = 51)	*P* value	Missing data
Age, y (mean; SD)	54.1 (12.7)	63.2 (12.7)	58.7 (13.4)	0.014	0
Gender, male (n; %)	15 (60)	21 (80)	36 (70.6)	0.104	0
Duration of HD, d (mean; SD)	144.6 (67.8)	145.8 (55.7)	145.2 (61.3)	0.065	0
Coronary calcification score (mean; SD)	102 (164.8)	2520.9 (1563.8)	1335 (1651)	<0.001	0
Calcium, mmol/L (mean; SD)	2.3 (0.2)	2.3 (0.3)	2.3 (0.2)	0.800	1
Phosphorus, mmol/L (mean; SD)	1.8 (0.4)	1.8 (0.6)	1.8 (0.5)	0.857	1
Albumin, g/L (mean; SD)	39.4 (2.9)	36.2 (5.2)	37.8 (4.5)	0.011	1
Alkaline phosphatase, U/L (mean; SD)	99.0 (51.9)	110.0 (44.5)	104.2 (48.2)	0.490	13
Glucose, mmol/L (mean; SD)	8.8 (7.2)	9.3 (6.9)	9.0 (7.0)	0.790	1
HbA1C (mean; SD)	0.07 (0.02)	0.07 (0.02)	0.07 (0.02)	0.716	28
C reactive protein, mg/L (mean; SD)	5.5 (8.2)	31.8 (46.1)	19.3 (35.7)	0.091	30
Intact PTH, pmol/L (mean; SD)	104.5 (129.0)	104.3 (83.0)	104.4 (108.8)	0.994	10
Uric acid, μmol/L (mean; SD)	380.4 (75.5)	372.8 (78.5)	376.6 (76.1)	0.750	9
URR (mean; SD)	65.4 (16.1)	66.2 (7.3)	65.8 (12.3)	0.834	2
Hemoglobin, g/L	112.76 (12.30)	119.69 (11.31)	116.29 (12.20)	0.041	0
Comorbidities					
COPD (n; %)	9 (36 %)	6 (23 %)	15 (29.4)	0.311	0
Liver disease (n; %)	2 (8 %)	0 (0 %)	2 (3.9)	0.141	0
Diabetes (n; %)	13 (52 %)	18 (69 %)	31 (60.8)	0.208	0
Hypertension (n; %)	23 (92 %)	24 (92 %)	47 (92.2)	0.967	0
Systolic blood pressure, mmHg (mean; SD)	147.6 (23.2)	145.4 (22.7)	146.5 (22.8)	0.737	0
Diastolic blood pressure, mmHg (mean; SD)	78.8 (15.9)	80.2 (14.2)	79.5 (14.9)	0.742	0
Baseline Cardiovascular Disease					
Myocardial infarction (n; %)	4 (16 %)	7 (27 %)	11 (21.6)	0.343	0
CABG (n; %)	2 (8 %)	3 (11 %)	5 (9.8)	0.671	0
Percutaneous coronary intervention (n; %)	1 (4 %)	5 (19 %)	6 (11.8)	0.091	0
Atrial fibrillation (n; %)	2 (8 %)	5 (19 %)	7 (13.7)	0.244	0
DVT (n; %)	5 (20 %)	0 (0 %)	5 (9.8)	0.016	0
Pulmonary embolism (n; %)	4 (16 %)	0 (0 %)	4 (7.8)	0.034	0
Cause of ESRD					
Glomerulonephritis (n; %)	11 (44 %)	2 (8 %)	13 (25.5)	0.003	0
Polycystic kidney disease (n; %)	2 (8 %)	1 (4 %)	3 (5.9)	0.529	0
Diabetes mellitus (n; %)	6 (24 %)	12 (46 %)	18 (35.3)	0.098	0
Hypertension (n; %)	5 (20 %)	10 (38 %)	15 (29.4)	0.148	0
Other (n; %)	1 (4 %)	1 (4 %)	2 (3.9)	0.977	0
Medications					
Calcium carbonate (n; %)	17 (68 %)	18 69 %	35 (68.6)	0.925	0
Sevelamer (n; %)	1 (4 %)	0 (0 %)	1 (2.0)	0.303	0
Bisphosphonates (n; %)	1 (4 %)	0 (0 %)	1 (2.0)	0.303	0
Warfarin (n; %)	4 (16 %)	5 (19 %)	9 (17.6)	0.762	0
Alfacalcidol (n; %)	14 (56 %)	9 (35 %)	23 (45.1)	0.125	0

*HD* hemodialysis, *CABG* coronary artery bypass grafting, *DVT* deep venous thrombosis, *ESRD* End-stage renal disease. Missing data 0-2 % with the exceptions of alkaline phosphatase (25 %), Hb:A1C ratio (55 %), C reactive protein (59 %), INR (29 %), iPTH (20 %), and uric acid (18 %)

**Fig. 1:**
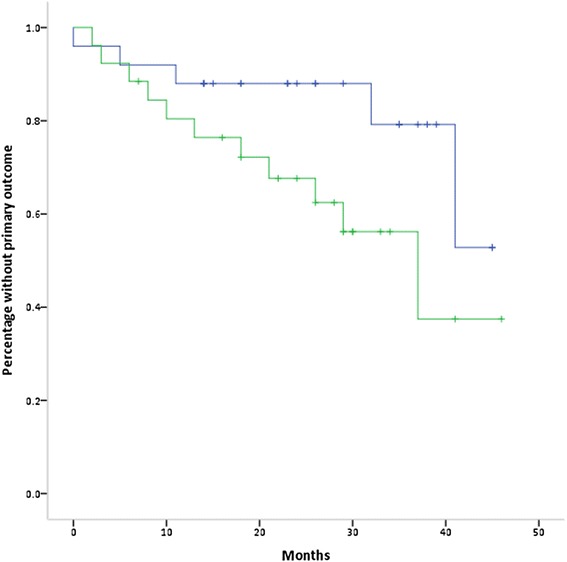
Kaplan Meier curve showing primary outcomes by coronary calcification

There were 26 primary outcomes (Table 2). Of these, 77 % of events occurred in the patients above the median coronary calcification score (20 outcomes in 586 patient months) compared with 23 % of events in the patients below the median (6 outcomes in 642 patient months) (*p* = 0.006). There were a total of 10 deaths: 8 in the group above the median and 2 in the group below the median (*p* = 0.04) (Table 3). Of these, 5 were cardiovascular deaths: 4 in the group above the median and 1 in the group below the median (*p* = 0.2). The 26 primary events occurred in 16 patients: 11/26 (42 %) of these patients were in the group above the coronary calcification median compared with 5/25 (20 %) in the group below the median (*p* = 0.086). By Cox regression, the hazard ratio for coronary calcification above, compared with below the median, was 2.5 (95 % CI 0.87, 7.3; *p* = 0.09) (Fig. 1). When adjusted for age, the hazard ratio was 1.7 (95 % CI 0.55, 5.4; *p* = 0.4). For myocardial infarction alone, the hazard ratios for above, compared with below the median, were 2.5 (95 % CI 0.65, 9.9; *p* = 0.2) and 1.7 (95 % CI 0.4, 7.3; *p* = 0.5), unadjusted and adjusted for age, respectively. For death, the hazard ratios were 4.6 (95 % CI 0.98, 21.96; *p* = 0.054) and 2.4 (95 % CI 0.45, 12.97, *p* = 0.3) respectively.

**Table 2:** Outcomes in the 51 patients over 1228 patient months

Outcome	Coronary calcification below median (n = 25)	Coronary calcification above median (n = 26)	Total (N = 51)	*P* value
Primary outcomes (n)	6	20	26	0.006^b^
Total patients with events (n)	5 (20 %)	11 (42 %)	16	0.086^a^
Myocardial infarction (n)	3	10	13	0.054^b^
Stroke (n)	1	2	3	0.58^b^
Death (n)	2	8	10	0.041^a^

^a^Chi squared

^b^Poisson

**Table 3:** Causes of death

Cause of death	Coronary calcification below median (n = 25)	Coronary calcification above median (n = 26)	Total (N = 51)	*P* value
Cardiovascular (n; %)	1 (4 %)	4 (15 %)	5	0.172
Other (n; %)	1 (4 %)	4 (15 %)	5	0.172
Death total (n; %)	2 (8 %)	8 (31 %)	10	0.041

## Discussion

The direction of our data suggests that coronary artery calcification may predict death, although this did not reach statistical significance. Additionally, there were tendencies towards association with future myocardial infarction, total events and event-free survival. Our findings suggest an increased risk of CV events, CV death, and death from all causes associated with having a higher coronary calcification score. When adjusted for age, the hazard ratio decreases from 2.5 (95 % CI 0.87, 7.3) to 1.7 (95 % CI 0.55, 5.4), suggesting that age may be a confounder.

Our results are consistent with one prospective study of 103 patients on HD with a follow up of 44 months that demonstrated a tendency towards increased relative risk for death of 2.7 (95 % CI 0.9, 8.3) for patients on hemodialysis having coronary calcification scores above 200 compared to those with scores below 200 (*p* = 0.08) [11]. Similarly, another prospective study examining 56 prevalent patients on HD over 15 months [8] found that patients with coronary calcification scores above the median had a relative risk for cardiovascular events of 2.6 (95 % CI 1.2, 5.5; *p* = 0.01), a relative risk for cardiovascular death of 3.1 (95 % CI 0.7, 14.3; *p* = 0.14), and a relative risk for death from all causes of 3.3 (95 % CI 1.4, 7.8; *p* = 0.007) [8]. However, this study used a composite for cardiovascular events that consisted of a heterogeneous mix of hard and soft outcomes including angina, myocardial infarction, percutaneous coronary intervention, CABG, severe valve disease, heart failure, and stroke [8]. In a 24-month prospective study of 117 patients with CKD who were not on HD, patients with coronary calcification scores ≥ 400 had an increased risk of cardiovascular events (a heterogeneous cluster of acute myocardial infarction, stroke, angina, arrhythmia, uncontrolled hypertension and cardiac failure) (HR = 3.53; 95 % CI: 1.03, 12.06; *p* = 0.04), and hospitalizations (HR = 4.05; 95 % CI 1.4, 11.5; *p* = 0.009) [12]. Cox regression was not conducted for deaths as there were no deaths in patients with coronary calcification scores < 400 compared with 4 in the group ≥ 400 (*p* = 0.002) [12]. Another study of 64 patients with ESRD on maintenance HD followed for 18 months found, in its secondary analysis, that cardiovascular outcomes occurred in 35 % of patients with a coronary calcification score > 10 compared, and in 0 % of patients with a coronary calcification score < 10 [10]. Patients who experienced a cardiovascular event had a higher mean coronary calcification score (1017 SD 975) than those who had not (129 SD 336) (*p* = 0.02) [10]. As in other studies, the cardiovascular outcome composite included both hard and soft outcomes (new-onset angina, myocardial infarction, angioplasty, and coronary artery bypass surgery) [10]. Another prospective study in 127 incident patients on HD followed for 18 months found a mean baseline coronary calcification score of 391 (SD 693) in patients alive at the end of the study and 1373 (SD 2034) in the 34 patients who had died (*p* <0.0001) [13]. Cardiovascular outcomes and risk were not reported [13].

One previous investigation of incident patients on HD reported a proportional increase in mortality with increasing coronary calcification, but did not report cardiovascular outcomes [14, 15]. This study, a clinical trial investigating the effect of the non-calcium phosphate binder sevalamer hydrochloride compared with calcium-containing phosphate binders on progression of coronary artery calcification in patients new to hemodialysis, observed progression of CAC in patients with baseline CAC >30, and that patients who were randomized to calcium-containing phosphate binders had a more rapid rate of progression that those randomized to sevalamer (*p* = 0.056 at 12 months; *p* = 0.01 at 18 months) [14].

Coronary calcification above the median was associated with older age, higher alkaline phosphatase, and higher hemoglobin, and a tendency toward a higher prevalence of diabetes as cause of ESRD (but not a higher prevalence of current diabetes). No differences were seen in serum calcium, phosphorus, calcium-phosphate product, iPTH, urea reduction ratio, or current warfarin use. Cross-sectional studies and baseline associations have shown consistent correlations between coronary calcification score and age [9, 11, 13, 18], duration of diabetes [6], HD vintage [7–9, 11], alkaline phosphatase [7], and fetuin-A [9]. Associations have been inconsistent for coronary calcification score and serum intact PTH [9, 10, 13], C-reactive protein [7, 9, 10, 13], serum calcium [7, 9, 18], serum creatinine [7, 13], cholesterol [7, 18], diabetes [7, 11, 18], smoking [7, 18], and hypertension [11, 18].

This study is one of the few that has looked at coronary artery calcification in incident ESRD, and is the only one, to our knowledge, that has prospectively examined both cardiovascular outcomes and death in this population. Much of the prior evidence has been derived from prevalent patients on maintenance HD [7–11, 18]. We had a well-characterized population at baseline, with an a priori decision to group patients on the basis of having a coronary calcification score above and below the median. Our composite outcome was a homogenous event cluster of serious, adjudicated cardiovascular events (stroke, MI), cardiovascular death, and death from all causes. We learned that patients at this stage of illness are very much deterred by the respondent burden of testing that requires an additional appointment. We were limited by a small sample size and a small number of events and thus, we were limited in the ability to perform multivariable adjustments. The small sample size may have led to type II error in the absence of association between biochemical markers, baseline clinical status and cardiac calcification. Patients who consented to this calcification substudy were younger than those in the study as a whole, and though this should not threaten the internal validity of the findings, it is a reminder of the challenges of observational studies with substantial respondent burden (such as the additional visit and risk associated with CT in this study) in patients already heavily burdened by an intrusive disease and an intrusive treatment.

## Conclusion

We conclude that, within the limits of our small sample size, our data are consistent with those of others showing that in incident patients with ESRD, coronary artery calcification predicts cardiovascular events and death. Coronary calcification was highly prevalent at baseline in this study, even though the patients were younger than the mean for patients starting dialysis in most of the developed world. Future research should aim to understand the mechanism of calcification in patients with chronic kidney disease before and after ESRD as well as evaluate therapeutic interventions to prevent coronary calcification or ameliorate its effects. We hope that this work will be a useful addition to future meta-analyses and will also help to provide information on baseline central tendencies and distributions to others who plan trials in this area. We also note that it may be challenging to recruit patients to studies that involve a special appointment in patients who have just started dialysis.
